# Supplementary material for: Repurposing Niclosamide to Modulate Renal RNA-Binding Protein HuR for the Treatment of Diabetic Nephropathy in db/db Mice
Source: Int J Mol Sci. 2024 Sep 6;25(17):9651. doi: 10.3390/ijms25179651 (PMC11394915; doi:10.3390/ijms25179651)
Supplement: Supplementary file 1 [file ijms-25-09651-s001.zip › ijms-3180134-supplementary.pdf]

Table S1,

Antibody resource and working solution information

Antibody name	Resource Company	Catalog number	Assay	Dilution fold
Mouse anti-HuR IgG	Santa Cruz Biotechnology Inc, Santa Cruz, CA, USA	sc-5261	IF/WB	1:100 /1:1000
Rabbit anti-Col-IV IgG	Rockland Immunochemicals Inc., Limerick, PA, USA	600-401-106	IF	1:150
Goat anti-Nephrin (G-20) IgG	Santa Cruz Biotechnology Inc.	sc-32530	IF	1:100
Rabbit anti-WT-1 (C-19) IgG	Santa Cruz Biotechnology Inc.	sc-192	IF	1:50
Rabbit anti- FN IgG	Sigma-Aldrich, Saint Louis, MO, USA	F3648	WB	1:1000
Mouse anti-a-SMA IgG	Sigma-Aldrich, Saint Louis, MO, USA	A5228	WB	1:2500
Mouse anti-NF-kBp65 IgG	Cell Signaling Technology.	6956S	WB	1:500
Mouse anti-gp91[phox] (Nox2) IgG1	BD Biosciences, San Jose, CA, USA	611414	WB	1:500
Rabbit anti-Angpt-1 IgG	EMD Millipore Corporation, Temecula, CA, USA	AB10516	WB	1:500
Rabbit anti-Angpt-2 IgG	EMD Millipore Corporation.	ABC882	WB	1:1000
Rabbit anti-Wisp1 IgG	Abcam, Cambridge, U.K.	Ab260036	WB	1:500
Mouse anti-ß-actin IgG	Sigma-Aldrich, Saint Louis, MO, USA	A5441	WB	1:2500
Goat anti-GAPDH IgG	GenScript, Piscataway, NJ, USA	A00191	WB	1:2500
Alexa Fluor™ 594-conjugated goat anti-mouse IgG (H+L)	Invitrogen, Carlsbad, CA, USA	A-11005	IF	1:200
Rhodamine Red™ -X-conjugated donkey anti-rabbit IgG	Jackson ImmunoResearch Laboratories Inc. West Grove, PA, USA	711-295-152	IF	1:200
Rhodamine Red™ -X-conjugated donkey anti-goat IgG	Jackson ImmunoResearch Laboratories Inc.	705-295-147	IF	1:200
Cy™3-conjugated donkey anti-rabbit IgG (H+L)	Jackson ImmunoResearch Laboratories Inc.	711-165-152	IF	1:200
HRP-conjugated donkey anti-rabbit IgG	Jackson ImmunoResearch Laboratories Inc.	711-035-152	WB	1:1000
HRP-conjugated goat anti-rabbit IgG	Jackson ImmunoResearch Laboratories Inc.	111-035-144	WB	1:1000
HRP-conjugated goat anti-mouse IgG	Jackson ImmunoResearch Laboratories Inc.	115-035-146	WB	1:1000
HRP-conjugated rabbit anti-goat IgG	Jackson ImmunoResearch Laboratories Inc.	305-035-003	WB	1:2000
4’, 6-diamidino-2 phenylindole (DAPI)-Fluoromount-G	Southern Biotech, Birmingham, Al, USA	0100-20	IF	No dilution

IF: immunofluorescent staining, WB: western blot assay
